# Supplementary material for: In Vitro Cytotoxic Effects and Mechanisms of Action of Eleutherine Isolated from Eleutherine plicata Bulb in Rat Glioma C6 Cells
Source: Molecules. 2022 Dec 13;27(24):8850. doi: 10.3390/molecules27248850 (PMC9785660; doi:10.3390/molecules27248850)
Supplement: Supplementary file 1 [file molecules-27-08850-s001.zip › molecules-2039183-supplementary.pdf]

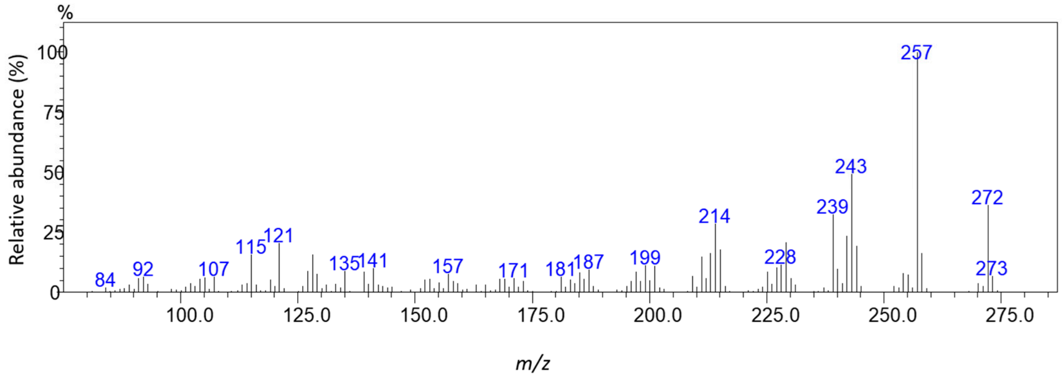

Figure S1. Spectrum of mass (70 eV) of eleutherine isolated from the bulbs of *Eleutherine plicata*.

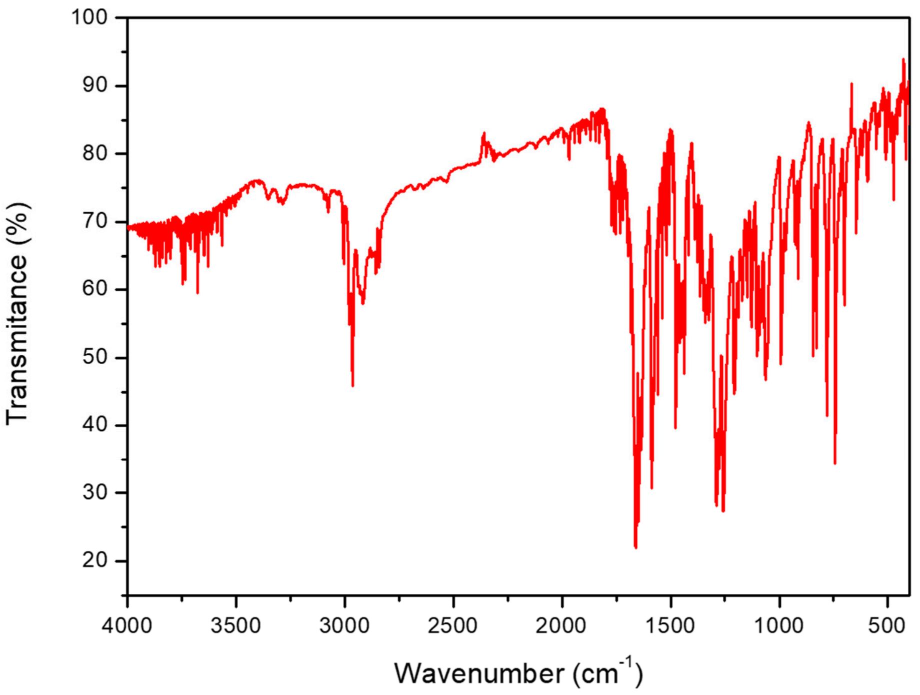

Figure S2. Spectrum of FT-IR (KBr) of eleutherine isolated from the bulbs of *Eleutherine plicata.*

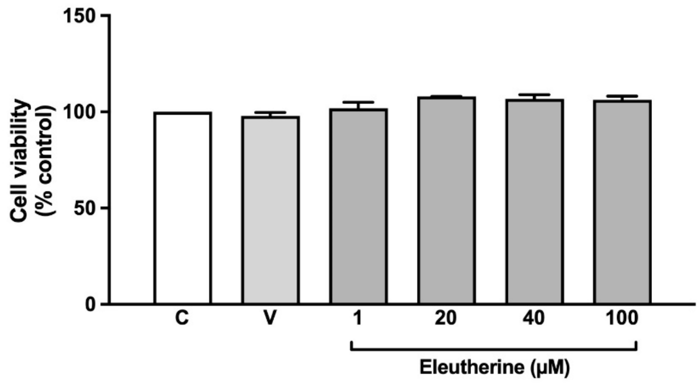

Figure S3: Eleutherine is nontoxic to nonneoplastic (glial) cells as analyzed by MTT assay. Cells were treated with different concentrations of eleutherine for 24 h. Data are presented as the mean ± SEM of three independent trials (ANOVA, Tukey post test). C, control; V, vehicle.
